# Bipolar Disorder XI: Continuous Decline of Lithium Response Inversely Correlated to Time Before Drug Administration in Relation to Onset of a Bipolar Disorder

**DOI:** 10.1016/j.bpsgos.2026.100791

**Published:** 2026-07-21

**Authors:** Per-Olof Nylander, Erik Lexne, Christer Lehman, Lars Brudin, Finn Bengtsson

**Affiliations:** a Linköping University, Linköping, Sweden; b Academic Clinic of Färjestaden, Färjestaden, Sweden; c Forensic Psychiatric Regional Clinic, Sundsvall, Sweden; d Department of Medical and Health Science, Linköping University, Linköping, Sweden; e Department of Biomedical and Clinical Sciences, Linköping University, Linköping, Sweden

**Keywords:** Bipolar disorder type I and II, Gradual decline in drug response, Lithium treatment, Long-term follow-up, Time as a predictor of lithium response, Years before lithium

## Abstract

**Background:**

Bipolar disorder (BD) is a severe disease affecting 1% of the general population. BD comprises recurrent depressive, hypomanic, and manic episodes. BD occurs as type I (BDI) and type II (BDII). In this study, we investigated the duration of illness before lithium treatment started in BDI and BDII.

**Methods:**

A long-term follow-up study of 192 patients with BD (diagnosed following DSM-IV criteria) were collected from lithium dispensaries. Patients with BDI and BDII were divided according to lithium response.

**Results:**

We found that patients with BD responded better to lithium if the treatment was introduced early after diagnosis compared with when treatment was started later in the course of the disease. From the diagnosis of BD, BDI was in general introduced earlier to lithium treatment compared with BDII. Logistic regression analysis showed that a continuous decline in lithium response was associated with the time lag before drug administration in relation to onset of the BD. To the best of our knowledge, we can for the first time report that lithium response is associated with a continuous decrease over time from the onset of BD until the drug treatment is started.

**Conclusions:**

A short duration of illness before introduction of lithium treatment is a predictor of better lithium response regardless of whether BDI or BDII is present. Lithium response seems to gradually decline with an increasing duration of illness before lithium treatment is started.

## Plain Language Summary

Bipolar disorder (BD) is a severe disease affecting approximately 1% of the general population. BD occurs as type I and II. A long-term follow-up study of up to 25 years of 192 patients with bipolar (DSM-IV) were collected from lithium dispensaries. BDI and BDII were divided according to the lithium response. A short duration of illness before the introduction of lithium treatment is a predictor of better lithium response regardless of whether BDI or BDII is present. Lithium response seems to gradually decline with an increasing duration of illness before lithium treatment is started.

Bipolar disorder (BD) is a common chronic affective disease, and approximately 1% of the general population is affected. BD is characterized by recurrent depressive, hypomanic, and manic episodes. The disorder is strongly associated with suicidal behavior such as suicidal thoughts, suicide attempts (SAs), and completed suicide (1, 2, 3, 4). Furthermore, BD also has an adverse impact on the quality of life (5,6).

BD can be divided into 2 subtypes: BDI and BDII. Both diagnoses have recurrent depressive episodes, but BDI also has both recurrent hypomanic and manic episodes, whereas BDII only has recurrent hypomanic episodes. It has previously been thought that BDI is a more severe form of BD; however, BDII has also been concluded to be a severe form of BD [for a review, see (7,8)]. Compared with BDI, BDII is characterized by a higher burden of depression, defined by the number and frequency of depressive episodes, severity, and duration of episodes. There are several studies concerning differences in the age of onset (AOO) between BDI and BDII; however, the results are heterogeneous and not conclusive [for a review, see (7,8)].

Patients with BD require long-term prophylactic treatment to prevent new episodes in the future. It is well known that lithium is effective in preventing depressive and especially manic episodes, acting as a mood stabilizer in BD (9, 10, 11, 12, 13, 14, 15). Meta-analysis of several controlled and open clinical trials of different treatment lengths has shown that lithium reduces recurrence of episodes in BD by approximately 29% (16,17). Lithium is also well known to prevent SAs and death by suicide in BD (18,19).

Treatment with lithium prophylaxis in BD shows that patients with BD respond differently and can therefore be divided into complete, partial, and non-responders. There are several studies of lithium response in BD, some over only a short period (20,21) and other studies with a longer follow-up period while on lithium (22, 23, 24). Altogether, the results are heterogeneous and vary between 17% to 40% for complete responders, 41% to 52% for partial responders, and 19% to 31% for nonresponders. The difference in follow-up time may explain the heterogeneous results because a longer observation time statistically increases the risk for a new relapse in the future.

It is important to predict early if a patient with BD will be a good or poor responder to lithium treatment. Patients with BD who may not respond or respond poorly to lithium can therefore instead be treated earlier with another mood stabilizer rather than lithium. Therefore, it is important to find clinical variables that can provide clues and be useful for clinicians to evaluate if a patient with BD will be a good or poor lithium responder.

Several predictors of lithium response have been suggested, for example, the course of the disorder, family history (FH) of BD, FH of lithium response, AOO, number of hospitalizations, mania-depression interval course sequence, depression-mania interval, continuous cycling, rapid cycling, BDII, pretreatment episodes, and episode sequence [for a review, see (25)]. In a meta-analysis, the authors suggested that predictors of good response are mania-depression interval sequence, absence of rapid cycling and psychotic symptoms, a short duration of the disorder before lithium treatment, FH of BD, and late AOO (25). Furthermore, they suggested that the number of episodes before lithium treatment, the number of hospitalizations before lithium treatment, and FH of lithium response may be related to good lithium response. However, yet another important observation was made in that the history of many previous episodes was associated with poor response to lithium or placebo but not to valproic acid (26).

Several studies have examined whether duration of illness before lithium treatment is a possible predictor of lithium response (21,27, 28, 29, 30, 31, 32, 33, 34, 35, 36). However, the studies have been performed using different methodological designs, which might be an explanation for the fact that the results are heterogeneous and not definitive. In a study of 52 patients with BD, it was found that a positive lithium response was associated with having a first treatment with lithium at the age of 20 years or earlier (27). Another study of 300 patients with BD (studied before lithium 10–12 years and while on lithium for at least 6 months) divided into 3 groups based on the Alda score (good, partial, and nonresponse) found no association between the duration of illness before lithium and lithium response (21). Furthermore, in a study of 300 patients with BD divided into 3 groups based on the Alda score (complete, partial, and nonresponse), it was found that a delayed introduction of lithium was associated with a poor response (28).

Another small (*N* = 61) long-term follow-up study of both before and while on lithium (mean 11 and 4.5 years, respectively) consisted of patients with BD or major depression (29). It was found that lithium responders were patients who were treated with lithium early in the course of the disorder. Furthermore, a second long-term follow-up study of both before lithium (mean 5 to >21 years) and while on lithium (4–8 years) consisted of 270 patients with BD or recurrent depression (30). Patients in this study were divided into the following groups: very early (within 5 years), early (within 10 years), late (11–20 years), and very late (≥21 years) depending on when lithium was introduced after onset. Best improvement while on lithium was found in patients introduced to lithium in the first 10 years after onset (very early and early groups).

A large register-based study of BD (*N* = 3762) has shown that increased rates of nonresponse were associated with the duration of illness before lithium (31). A second large register-based study (*N* = 4714) also found that initiation of lithium treatment early following first psychiatric contact or a single manic or mixed episode is associated with an increased possibility of lithium response (32).

There are only 4 long-term follow-up studies of patients with BD exclusively both before and while on lithium; however, these studies followed different methodological designs (33, 34, 35, 36). The first 2 long-term follow-up studies (33,34) of BD found no association between the duration of illness before lithium treatment and lithium response. The first study of 118 patients with BD with a follow-up period both before and while on lithium (mean 8.4 and 11.1 years, respectively) divided the patients in 2 groups based on the median frequency of relapses and studied continuous measures in relation to the number of relapses per year while on lithium (33). The second study of 111 patients with BD with a follow-up period both before and while on lithium (mean 8.4 and 14.2 years, respectively) was based on a comparison between excellent responders, partial responders, and nonresponders (34).

The third long-term follow-up study of 317 patients with BD both before and while on lithium (mean 8 and 6 years, respectively) studied the differences in frequency and duration of affective episodes as well as hospitalizations between the 2 periods. It was found that starting lithium treatment early predicted better improvement while on lithium (35). The fourth study of 360 patients with BD both before and while on lithium (mean 8 and 6 years, respectively) found that patients with a superior treatment response of lithium (>75% improvement) was associated with a shorter time period between the onset of the disorder and introduction of lithium treatment (36). Furthermore, they also found that in a median split of high versus low improvement, a higher percentage of improvement was associated with a shorter time from onset of the disorder to initiation of lithium treatment.

Altogether, there are 4 long-term follow-up studies based exclusively on patients with BD that have studied periods both before initiation of lithium treatment and while on lithium with the aim to investigate if duration of illness before lithium is associated with lithium response (33, 34, 35, 36). Two of 4 studies have found a positive correlation between duration of illness before lithium and lithium response, and the remaining 2 studies did not. None of the previous studies based exclusively on patients with BD have looked for whether there is a continuing decline in lithium response with an increase in duration of illness before introduction of lithium (33, 34, 35, 36). Therefore, in our opinion, there is still a need for more long-term follow-up studies both before and with an especially long observation time while on lithium to investigate if a clinical variable such as duration of illness before lithium treatment starts can be associated as a crucial predictor for the overall lithium response in cases of BD.

The main aims of this study were, therefore, to first assess whether the duration of illness before introduction of lithium treatment is associated with the degree of lithium response in BD. Second, if so, we wanted to assess if such a decline in lithium treatment response appears in a gradual manner over time from the onset of a BD. The latter observation could provide important information to clinicians to carefully evaluate if BD is at hand or not, and if so, that the introduction of lithium treatment is started as early as possible. To investigate these matters, we used a very long-term follow-up study of 192 patients with BD sampled from lithium dispensaries in Sweden. All patients were studied both before and while on lithium treatment.

## Methods and Materials

One hundred ninety-two patients with BD were recruited from lithium dispensaries of Umeå, Sundsvall, and Härnösand Hospitals in northern Sweden. Before assessing the material, it was compared for age, gender, and number of BDI and BDII, respectively, from each dispensary. It was found that although the 3 dispensaries included a different number of patients (*n* = 106, 52, and 34, respectively), none of the parameters above altered significantly between investigation sites, thus allowing for pooling the patient material into one cohort.

Patients with diagnoses of alcoholism, drug abuse, epilepsy, or mental retardation were excluded. For all included patients, medical records containing previous psychiatric illness, episodes, AOO, FH, and medical treatment were available. All patients were continuously followed from the date of onset of BD, and all episodes were documented by experienced psychiatrists. Furthermore, for all patients, the BD diagnoses and all other data were scrutinized and confirmed by 2 authors (CL and P-ON, as experienced psychiatrists by training). This process also included interviews using Present State Examination (PSE-10) (37) and checking all patients’ data by applying the Operational Criteria Checklist (OPCRIT) (38). PSE-10 and OPCRIT were performed once by one of the authors (CL or P-ON), and the data obtained were used to confirm the diagnosis. Interrater reliability (IR) was 1.0 concerning diagnoses, episodes, AOO, and all other clinical variables. This very good precision in IR was probably based on the experienced psychiatrists CL and P-ON working in parallel at the same clinic for many years. Data sampling was performed in the past 2 years of the project. The interviews were conducted when all data were sampled for the study at the end of the follow-up study (past 2 years).

DSM-IV criteria (39) were used for diagnosing BDI and BDII. An affective episode was defined according to DSM-IV and Research Diagnostic Criteria (40) for major depressive, hypomanic, or manic episode, requiring hospitalization or treatment as an outpatient. AOO was defined by the first episode of depression, hypomania, or mania. We also defined whether the first episode was depression, hypomania, or mania. All episodes of depression, hypomania, and mania were plotted in a lifetime diagram from AOO to the end of the study, which made it possible to determine when each episode occurred. For each patient, information was obtained regarding the total number of episodes and frequency of depression, hypomania, and mania from AOO to end of study. The number of years from AOO until criteria for diagnoses of BD was also recorded.

Data concerning lithium treatment was divided into length of time before lithium treatment was introduced and when on/off lithium therapy occurred. This enabled a possibility to measure episode frequency before initiation of lithium and the effect of on/off lithium dispensation. Treatment response was calculated by comparing the number of episodes per year before and while on lithium.

When calculating lithium response, we used 2 methods. First, the median response of lithium treatment of 68% was used as a divider by denoting less response as ≤68% or a better response as ≥68%. Second, to establish whether the change in lithium response over time was to be considered continuous or not, regression analyses were performed (both univariate and multivariate, see statistics below).

Compliance was defined by measurement of serum lithium (S-lithium) levels (in blood samples) and medical records. Poor compliance was defined when notes in the medical record reported discontinuation of medication or S-lithium levels were inadequately low or were fluctuating over time. The latter is defined when S-lithium displayed a significant variation despite an unchanged dose of lithium or if a change in lithium dose was not followed by an expected change in concentration of S-lithium. The usual therapeutic interval for S-lithium for most patients was between 0.5 and 0.9 mmol/L, referring to trough levels (i.e., sample drawn before next dose) at steady state (usually achieved after 1 week of daily intake of the drug). The treatment goal was to have the optimal effect on as a low dose as possible. S-lithium levels above 1.2 mmol/L increase the risk for intoxication, and levels above 3.0 mmol/L are considered as life threatening and potentially lethal.

FH was obtained in detail from medical records by semistructured interviews of patients and relatives by 2 authors (experienced psychiatrists CL and P-ON) and using the Family History Research Diagnostic Criteria (41) for definition of FH of affective disorder (AD) (major depression, recurrent depression, and BDI and BDII) and FH of suicide. Only patients with an FH of AD among first- and/or second-degree relatives were defined as having an FH of AD. Altogether, we found 418 first-degree (*n* = 242) or second-degree (*n* = 176) relatives with an AD. Patients with an FH of other psychiatric disorders than AD were not included in this study to avoid a heterogeneous background. Patients were divided into 2 groups, with or without a FH of AD. It was also recorded whether patients had performed single or multiple SAs; however, these data were not analyzed further in this investigation.

Before initiation of lithium treatment, a depressive episode was treated with antidepressants and, if needed, in combination with hypnotics and anxiolytics. In all patients, medication was registered in medical records at the psychiatric clinic. When lithium treatment started, lithium treatment was used alone and not in combination with any other psychotropic drug, except for hypnotics and anxiolytics when clinically motivated.

Statistics were calculated with SPSS version 23 and Statistica version 13.5.0.17 (TIBCO Software, Inc.). In addition, χ^2^ with Fisher’s exact test (two-tailed) was used for analysis of prevalence based on categorical variables. For comparisons of continuous variables, Student *t* test (normal distribution) and Mann-Whitney *U* test (non-normal distribution) were used. The length of time before lithium was categorized in quartiles, and these 4 groups were used as a continuous predictor in the logistic regression model. Similarly, the total observation time (before and after lithium) was categorized in quartiles and analyzed in the same way. These were then adjusted for gender and age (at onset of illness and at follow-up). This approach is used to assess how the risk of the dependent binary outcome changes across different ranges of the independent variable. The significance level was chosen at *p* < .05. The possibility of the occurrence of multiple significant errors was considered but chosen not to be adjusted for.

The Ethical Committee in Umeå (Sweden) approved this study (Dnr. 92-045 owned by the author P-ON).

## Results

Clinical data of 192 patients with BD were divided into 2 groups depending on the type of BD (BDI and BDII); the total sample of patients is provided in Table 1. The mean number of years before introduction of lithium treatment was significantly higher (*p* = .003) in patients with BDII compared with patients with BDI. Furthermore, the mean number of episodes before lithium treatment was significantly higher (*p* < .001) among patients with BDII compared with patients with BDI. The number of years on lithium treatment was significantly longer (*p* = .008) among patients with BDI compared with patients with BDII.

**Table 1:** Clinical Data of 192 Patients With BD Divided Into 2 Groups Depending on Type of BD (i.e., Type I and II) in a Long-Term Follow-Up Study of Approximately 25 Years

Variables	BD I, *n* = 156	BD II, *n* = 36	Total, *N* = 192	*p* Value
Gender, *n* (%)				
Female	94 (60.3%)	18 (50.0%)	112 (58.3%)	.268
Male	62 (39.7%)	18 (50.0%)	80 (41.7%)	
Current Age, Years				
Mean (SD)	56.6 (15.4)	59.3 (13.1)	57.1 (15.0)	
Median (range)	58.5 (22.0–87.0)	62.0 (34.0–79.0)	59.0 (22.0–87.0)	.326
AOO, Years				
Mean (SD)	30.5 (11.1)	32.6 (9.9)	30.8 (10.9)	
Median (range)	29.0 (15.0–63.0)	33.5 (14.0–58.0)	30.0 (14.0–63.0)	.148
Years Before Lithium				
Mean (SD)	10.6 (11.0)	15.4 (10.7)	11.5 (11.1)	
Median (range)	6.3 (0.5–50.0)	14.8 (0.5–40.0)	8.0 (0.5–50.0)	.003^a^
Episodes Before Lithium				
Mean (SD)	6.8 (6.6)	10.6 (5.2)	7.5 (6.5)	
Median (range)	5.0 (1.0–57.0)	10.0 (3.0–29.0)	6.0 (1.0–57.0)	<.001^a^
Episodes per Year Before Lithium				
Mean (SD)	1.3 (1.3)	1.1 (1.1)	1.3 (1.2)	
Median (range)	1.0 (0.2–8.0)	0.7 (0.2–6.0)	1.0 (0.2–8.0)	.390
Years on Lithium				
Mean (SD)	13.4 (8.2)	9.5 (7.3)	12.6 (8.2)	
Median (range)	12.0 (1.0–29.0)	7.0 (2.0–25.5)	11.0 (1.0–29.0)	.008^a^
Episodes on Lithium				
Mean (SD)	4.9 (5.3)	3.3 (3.4)	4.6 (5.0)	
Median (range)	3.0 (0.0–31.0)	2.0 (0.0–15.0)	3.0 (0.0–31.0)	.204
Episodes per Year on Lithium				
Mean (SD)	0.4 (0.4)	0.5 (0.5)	0.4 (0.4)	
Median (range)	0.3 (0.0–2.4)	0.3 (0.0–2.3)	0.3 (0.0–2.4)	.563
S-Lithium During Episode, mmol/L				
Mean (SD)	0.61 (0.13)	0.59 (0.09)	0.60 (0.13)	
Median (range)	0.60 (0.24–1.0)	0.60 (0.40–0.74)	0.60 (0.24–1.0)	.514
S-Lithium Without Episode, mmol/L				
Mean (SD)	0.62 (0.11)	0.58 (0.10)	0.61 (0.11)	
Median (range)	0.63 (0.30–0.89)	0.60 (0.40–0.79)	0.62 (0.30–0.89)	.089

*p* Values are obtained from Mann-Whitney’s *U* test or Fisher’s exact test for gender.

AOO, age of onset; BDI, bipolar disorder type I; BDII, bipolar disorder type II; S-lithium, serum lithium.

a Significant difference.

Table 2 shows clinical data in patients with BD divided into 2 groups depending on the degree of lithium response (defined by the median value of 68%). The mean number of years before lithium treatment was significantly higher (*p* < .001), and the mean number of episodes per year was significantly lower (*p* < .001) in patients with BD with a poorer lithium response (≤68%). The mean number of episodes per year before lithium treatment was lower in patients with BD with a poorer lithium response due to a longer time period before lithium treatment was initiated. Furthermore, the mean number of episodes as well as episodes per year while on lithium treatment were significantly higher (*p* < .001) in patients with BD with a poorer lithium response (≤68%).

**Table 2:** Clinical Data of 192 Patients With BD Divided Into 2 Groups Depending on Lithium Response (Defined by the Median Value of 68%)

Variables	Lithium Response		Total, *N* = 192	*p* Value
	≤68%, *n* = 95	>68%, *n* = 97		
BD, *n*(%)				
BDI	75 (79%)	80 (82%)	155 (81%)	
BDII	20 (21%)	17 (18%)	37 (19%)	.586
Gender, *n* (%)				
Female	54 (57%)	58 (60%)	112 (58%)	.770
Male	41 (43%)	39 (40%)	80 (42%)	
Current Age, Years				
Mean (SD)	57.2 (15.6)	57.0 (14.4)	57.1 (15.0)	
Median (range)	59.0 (22.0–86.0)	59.0 (22.0–87.0)	59.0 (22.0–87.0)	.859
AOO, Years				
Mean (SD)	29.5 (10.3)	32.2 (11.3)	30.8 (10.9)	
Median (range)	28.0 (14.0–62.0)	32.0 (15.0–63.0)	30.0 (14.0–63.0)	.105
Years Before Lithium				
Mean (SD)	13.8 (11.6)	9.3 (10.1)	11.5 (11.1)	
Median (range)	11.0 (1.0–50.0)	5.0 (1.0–50.0)	8.0 (1.0–50.0)	<.001^a^
Episodes Before Lithium				
Mean (SD)	8.2 (7.2)	6.8 (5.8)	7.5 (6.5)	
Median (range)	6.0 (1.0–57.0)	5.0 (1.0–42.0)	6.0 (1.0–57.0)	.094
Episodes per Year Before Lithium				
Mean (SD)	1.0 (0.9)	1.6 (1.4)	1.3 (1.2)	
Median (range)	0.7 (0.2–4.0)	1.2 (0.2–8.0)	1.0 (0.2–8.0)	<.001^a^
Years Before Lithium and Time on Lithium				
Mean (SD)	26.3 (12.0)	24.1 (12.6)	25.1 (12.3)	
Median (range)	25.0 (4.5–56.0)	23.0 (3.5–54.5)	24.0 (3.5–56.0)	.182
Years While on Lithium				
Mean (SD)	11.4 (7.4)	13.9 (8.7)	12.6 (8.2)	
Median (range)	10.0 (1.0–29.0)	13.0 (1.0–29.0)	11.0 (1.0–29.0)	.072^b^
Episodes While on Lithium				
Mean (SD)	6.6 (5.4)	2.6 (3.6)	4.6 (5.0)	
Median (range)	5.0 (1.0–31.0)	1.0 (0.0–18.0)	3.0 (0.0–31.0)	<.001^a^^,^^c^
Episodes per Year While on Lithium				
Mean (SD)	0.68 (0.48)	0.18 (0.21)	0.43 (0.44)	
Median (range)	0.55 (0.13–2.40)	0.11 (0.00–1.00)	0.32 (0.00–2.40)	<.001^a^^,^^c^
S-Lithium During Episode, mmol/L				
Mean (SD)	0.61 (0.12)	0.59 (0.14)	0.60 (0.13)	
Median (range)	0.60 (0.31–0.93)	0.58 (0.24–1.00)	0.60 (0.24–1.00)	.282
S-Lithium Without Episode, mmol/L				
Mean (SD)	0.61 (0.11)	0.61 (0.11)	0.61 (0.11)	
Median (range)	0.62 (0.31–0.89)	0.62 (0.30–0.87)	0.62 (0.30–0.89)	.848

*p* Values are obtained from Mann-Whitney’s *U* test for continuous variables or Fisher’s exact test for categorical variables.

AOO, age of onset; BD, bipolar disorder; BDI, bipolar disorder type I; BDII, bipolar disorder type II; S-lithium, serum lithium.

a Significant difference.

b Incorporated in the follow-up years (included in the multivariate logistic regression, and therefore not included separately in the multivariate logistic regression).

c Low *p* values are expected from the definition of the response variable and are not included in the multivariate logistic regression.

Univariate and multivariate logistic regression analysis is provided in Table 3. Both univariate (*p* = .004) and multivariate (*p* = .005) logistic regression analysis showed that lithium response (successful outcome) is significantly correlated with duration of years of illness before introduction of lithium treatment. A short duration of years of illness before the introduction of lithium treatment is correlated with a better lithium response. The best lithium response is found in the first quartile, and thereafter lithium response successively diminishes, with the poorest lithium response in the fourth quartile.

**Table 3:** Results From Follow-Up, Categorized as Successful (>68% Improvement Used as Median Value) or Not, and Analyzed Using Logistic Regression, Univariate and Multivariate Adjusted for Gender, Age, and Total Observation Time

Parameters	Total	*n* (%)	Univariate		Multivariate	
			OR (95% CI)	*p* Value	OR (95% CI)	*p* Value
Gender						
Female	112	58 (51.8%)	1.00	.679	1.00	.486
Male	80	39 (48.8%)	1.13 (0.63–2.01)		1.24 (0.68–2.27)	
Current Age, Years						
<60	101	52 (51.5%)	1.00	.779	1.00	.884
≥60	91	45 (49.5%)	0.92 (0.52–1.63)		0.94 (0.42–2.13)	
AOO, Years						
<30	93	41 (44.1%)	1.00	.087	1.00	.201
≥30	99	56 (56.6%)	1.65 (0.93–2.93)		1.54 (0.79–3.00)	
Diagnosis						
BDI	156	81 (51.9%)	1.00	.421		
BDII	36	16 (44.4%)	0.74 (0.36–1.54)			
Years Before Lithium and While on Lithium						
<14	49	28 (57.1%)	1.00	.370	1.00	.288
14–22	47	22 (46.8%)	0.89 (0.69–1.15)		1.24 (0.83–1.83)	
22–33	48	25 (52.1%)	0.79 (0.48–1.32)		1.53 (0.70–3.35)	
>33	48	22 (45.8%)	0.71 (0.33–1.52)		1.89 (0.58–6.14)	
Years Before Lithium						
<3	48	33 (68.8%)	1.00	.004^a^	1.00	.005^a^
3–8	46	23 (50.0%)	0.69 (0.53–0.89)		0.61 (0.43–0.86)	
8–17	45	20 (44.4%)	0.47 (0.28–0.79)		0.37 (0.18–0.74)	
>17	53	21 (39.6%)	0.32 (0.15–0.70)		0.23 (0.08–0.64)	
Episodes Before Lithium						
<3.5	48	26 (54.2%)	1.00	.087		
3.5–6	61	35 (57.4%)	0.60 (1.03–0.49)			
6–10	43	21 (48.8%)	0.37 (1.07–0.24)			
>10	40	15 (37.5%)	0.22 (0–0)			

AOO, age of onset; BDI, bipolar disorder type 1; BDII, bipolar disorder type II; OR, odds ratio.

a Significant difference.

## Discussion

Several clinical factors have been suggested to be a predictor of good response in BD to lithium treatment [for a review, see (25)]. One of these factors is a short duration of the disorder before lithium treatment is instituted.

In line with this notion, the most important observation made in this investigation is referred to in Tables 2 and 3. Hence, to the best of our knowledge, this is the first time in the literature regarding the overall response to lithium treatment in BD that a continuous worsening of the lithium response over time seems to be present. This continuing loss of lithium effect over time is evidenced by the logistic regression analyses seen in Table 3. This general worsening of the lithium response is inversely related to when the drug is introduced after onset of the BD.

We have clearly shown that this overall lithium effect is associated with a gradual decline over time from when the drug was introduced to patients with BD. Previous studies (33, 34, 35, 36) investigating patients with BD after onset of illness and after introduction of lithium have been able to analyze the drug response. These studies have followed patients exclusively affected by BD both before lithium for a period of approximately 8 years and while on lithium for a period between 5 and 11 years. In our study, we also followed patients with BD before lithium (10–20 years) and while on lithium (8–13 years). Our study is comparable with these studies because we also included patients exclusively affected by BD, but our study has the advantage of having an even longer total follow-up time of up to 25 years. However, 2 of these studies (33,34) could not find any changes in the lithium response over time in their parameters measured. The other 2 studies (35,36), showed similar findings to our study findings and were able to display a change with less lithium effect in relation to whether the drug was introduced very early or very late after onset of the illness. However, in these latter investigations (35,36), they only studied their parameters at 2 end points after introduction of the lithium treatment in BD. The more precise delineation of how the overall lithium response develops over time from onset of the BD has therefore not been possible to discern in these investigations because of their designs (35,36). With only 2 end points to establish the response to lithium treatment in studies such as these, they cannot tell us if the change in response to treatment does this in a gradual manner or whether it has a rapid change in the response at a certain time point related to the duration before treatment starts in relation to the onset of the BD. Although the everyday clinical reality presents a mix of patients not only affected by BD, it is also important in a scientific context to purify the patient sample to only include true patients with BD to be able to clearly state if the effect is associated solely with this disorder and not with a great variety of other comorbid conditions.

Some of the previous studies are register-based studies (31,33), some studies have been performed on small samples (27,29), some have mixed samples of BD and unipolar disorders (29,30), and some have a shorter observation time (6 months to 4 years and 6 months) while on lithium (21,27,29,30), or the treatment duration with lithium is not available (28). It is known that lithium treatment is efficient in BD in general and especially manic episodes (9, 10, 11, 12, 13, 14, 15). It has also been shown that lithium is more efficient in the treatment of depressive episodes among patient with BD compared with those with unipolar disorders [for a review, see (42)]. Therefore, it is not possible to calculate responders versus nonresponders in a heterogeneous patient group consisting of both BD and unipolar disorder. In a 5-year follow-up study, it was shown that while on lithium treatment, the individual number of complete responders decreased from 83% during the first year, 52% after 3 years, and 37% after 5 years (43). Therefore, by only presenting data based on a 5-year follow-up period, it is not valid to make a conclusion on how the regression of the response to drug treatment occurs. Whether it is a continuous and gradual response in these studies, or whether it is a sudden decline at any time point registered for starting lithium treatment after onset of the illness, is not feasible to state in these studies. Moreover, to make such statements, studies of this kind need, by necessity, to have a much longer follow-up period including data for both before and after lithium treatment has been introduced.

The importance of our present finding is that it supports the general context of a decline in lithium response related to the lag in time from the onset of BD to the start of treatment. It also provides evidence that this phenomenon occurs in a continuous fashion. This latter unique observation in our study provides for some speculations on how lithium is acting in BD. For example, what may be the reason for the response to successively subside over time from when lithium is introduced to a patient with BD after the onset of BD?

Lithium is a unique agent that has shown good clinical results as a treatment, being an antisuicidal agent, and has proven to serve as excellent prophylaxis in BD for over half a century (43). Although we know that lithium exerts a wide range of effects on neurotransmitters, intracellular pathways, mitochondrial function, and inflammatory regulation, none of these parameters in the mode of action of lithium has yet been concluded as causal for how lithium acts to the benefit of counteracting BD (44). However, with our novel findings of an association of a continuous decline in the general response to lithium over time from the onset of BD, it is possible to suggest a tentative explanation for how the phenomenon evolves and how it might be related to the mode of action of lithium on a basic molecular level.

Could it be that the prolongation of time of BD without lithium treatment from the time of onset of a BD will have the untreated disease damage the brain in such a manner that the drug efficacy subsequently declines? In such a case, could the disease perhaps make a cellular impact on the brain parenchyma that attenuates the drug effect exerted by lithium? If so, do the extra- or intracellular events where lithium acts on the brain to alleviate symptoms of the disease become increasingly insufficient? A parallel to both unipolar depression and psychotic disorder treatments can be made, in that an untreated patient with depression or psychosis patient will have less effect of an antidepressant or antipsychotic agent, respectively, the later this treatment is instituted after a depressed or psychotic episode occurs. It has been shown that frequent recurrent depressive episodes entail a higher risk for a new episode to occur, pronounced severity, shorter duration to next episode, chronic development, and less effect of antidepressive drugs in the disorder (45). Furthermore, some evidence also suggests that relapses in a psychotic disorder may be a critical factor in that illness progression contributes to morbidity and emergent treatment failure over time (46). Therefore, preventing relapses in early treatment also in a psychotic disorder is a long-term maintenance treatment in this illness (46).

This study also provides evidence that patients with BDII are more often associated with lithium treatment being introduced later in the course of the disorder. This could possibly result in a low lithium response in patients with BDII. However, it should be noted that although patients with BDII are most often treated significantly later after diagnosis than the patients with BDI, we could not establish, by the present data, that the patients with BDII themselves individually respond less favorably to lithium treatment than the patients with BDI. Thus, only the diagnosis of patients with BDI and BDII is probably not a useful clinical discriminator for identifying which patients with BD will respond well to lithium treatment. In summary, the most important clinical variable in all cases of BD seems, in our mind, to be the duration of illness before lithium treatment is initiated.

This investigation suffers from major limitations. The first and foremost limitation is that this is a retrospective study, limiting the strength of the evidence to merely being associative rather than conclusive for establishing a true causal relationship between the findings of BD, both in type I and II. Second, based on our findings associated with the diminishing response to lithium treatment related to the time lag from when it is instituted, our suggested ideas about the mode of action of the drug are, at best, very speculative. Nevertheless, accepting that we must limit our more generalized speculations based on previous investigations and data from this study, we would still like to propose the following argumentation. First, it is likely that the longer a core psychiatric condition such as BD is untreated, the worse biological damage to the brain may develop. Second, this, in turn, may be the reason why the appropriate drug treatment in this condition will act less well due to that the target for the drug treatment, i.e., the brain tissue on a cellular level, is less responsive as time goes by, causing the mechanism by which the drug exerts its effect to be hampered by the ongoing continuous changes occurring in the cells in the brain. Third, if such biological changes occur in the brain over time, maybe the doses and levels of S-lithium should be revised to suggest higher levels to meet a lesser pharmacological activity of the drug, or that co-medication with yet another mood-stabilizing drug could be introduced together with the lithium treatment, or that lithium is not chosen in favor of introducing another mood-stabilizing drug alone instead? Fourth, why differences in the overall response between BDI and BDII are difficult to find may result from variations found in the 2 conditions. Although patients with BDII may have lithium introduced too late to have an optimal effect, the patients with BDI may experience more brain damage caused by manic episodes. In summary, therefore, the 2 negative effects of either late introduction of lithium or manic damaging effects on the brain even out to a rather similar overall effect of lithium treatment in both situations.

## Supplementary Material

Key Resources Table
